# Impact of local composition on the energetics of *E*-centres in Si_1−*x*_Ge_*x*_ alloys

**DOI:** 10.1038/s41598-019-47385-0

**Published:** 2019-07-26

**Authors:** Stavros-Richard G. Christopoulos, Navaratnarajah Kuganathan, Alexander Chroneos

**Affiliations:** 1 Faculty of Engineering, Environment and Computing, Coventry University, Priory Street, Coventry, CV1 5FB United Kingdom; 2 Department of Materials, Imperial College London, London, SW7 2AZ United Kingdom

**Keywords:** Electronic devices, Computational chemistry

## Abstract

The energetics of the defect chemistry and processes in semiconducting alloys is both technologically and theoretically significant. This is because defects in semiconductors are critical to tune their electronic properties. These processes are less well understood in random semiconductor alloys such as silicon germanium as compared to elementary semiconductors (for example silicon). To model the random silicon germanium alloy we have employed density functional theory calculations in conjunction with the special quasirandom structures model for different compositions. Here we show that, the energetics of substitutional phosphorous-vacancy pairs (*E*-centres) in Si_1−*x*_Ge_*x*_ alloys vary greatly with respect to the local Ge concentration and the composition of the alloy. The most energetically favourable *E*-centres have a Ge atom as a nearest neighbour, whereas the dependence of the binding energy of the *E*-centres with respect to alloy composition is non-linear.

## Introduction

The substitution of the silicon native oxide with high-k dielectrics has enabled the replacement of silicon (Si) with materials such as germanium (Ge) or silicon germanium (Si_1−*x*_Ge_*x*_), which have better materials properties (e.g. higher mobilities)^1–10^. Si_1−*x*_Ge_*x*_ is a random alloy in the sense that two atom species can occupy one lattice site. This in turn can lead to Si-rich and Ge-rich regions that can impact the energetics of point defects. This has been previously demonstrated in Si_1−*x*_Ge_*x*_ alloys where there is a compositional dependence of self-diffusion (refer to^3,6,11^ and references therein) but also oxides where local compositional variation impacts diffusion^12,13^. Recently, Saltas *et al*^11^. studied self-diffusion in Si_1−*x*_Ge_*x*_ alloys as a function of temperature and Ge concentration within the *cBΩ* thermodynamic model^14–17^. In this study considerable deviations from linearity of the activation energies with respect to compositions were observed^11^. This non-linear behaviour was attributed to the diverse behaviour of the bulk properties of Si and Ge and is consistent with the experimental data.

The theoretical investigation of random alloys using advanced techniques such as density functional theory (DFT) is not trivial as it requires a very extensive number of calculations in large supercells. To reduce the supercell size and the number of calculations methods such as the special quasirandom structures (SQS) have been previously proposed^18^ and implemented in technologically important random alloys (more details are given in what follows)^19–23^. Considering group IV random alloys previous work has investigated the defect processes in Si_1−*x*_Ge_*x*_, Sn_1−*x*_Ge_*x*_ and Si_1−*x*−*y*_Ge_*x*_Sn_y_ alloys^24–28^.

The main aim of the present study is to use extended SQS cells in conjunction with systematic DFT calculations to investigate the energetics of substitutional phosphorous-vacancy pairs (*E*-centres) in Si_1−*x*_Ge_*x*_ alloys (x = 0.125, 0.25, 0.375, 0.5, 0.625, 0.75, 0.875). Particular emphasis will be placed on the effect of the composition of the alloy as well as the nearest neighbor environment to the defect.

## Results and Discussion

### Si_1−x_Ge_x_ structure and *E*-centres

Typical DFT calculations involve the construction of a supercell with periodic boundary conditions and as such they are immediately applicable for perfectly ordered structures. The situation is not straightforward, however, for disordered random alloys. Brute force methods (i.e. construct a very large supercell and randomly inserting the host atoms) is practically unfeasible for DFT as it necessitates very large supercells to reproduce the statistics of random alloys. Conversely, the SQS approach efficiently mimics the statistics of random alloys using small supercells and therefore it is a method that is compatible with DFT particularly when numerous defect calculations are required^18–23^. The qualitative difference of the SQS is that they are designed small-unit-cell periodic structures that mimic the most relevant near neighbour pair and multisite correlation functions of the random substitutional alloys. Their atomistic nature ensures that there is a distribution of distinct local environments that also exist in real random alloys. For the Si_1−*x*_Ge_*x*_ alloys considered the Si or Ge atoms can be surrounded by various Si_n_Ge_4−n_ coordination shells (n ranging from 0 to 4). This forms a distribution of local environments, which in turn influence the formation and energetics of dopant-defect interactions such as the *E*-centre^29^. The efficacy of the SQS technique to model random alloys has been demonstrated previously in numerous systems including group IV alloys^5,20,28^, oxides^21,30^, and III-V alloys^22,31^.

The *E*-centre in Si or Ge is a substitutional donor atom (for example P, As, Sb) bound with a nearest neighbor vacancy. The structure and energetics of *E*-centres in Si or Ge have been extensively investigated using both experimental and theoretical techniques^32–36^. Here as a criterion for the formation of the *E*-centre we consider the binding energies. A defect pair such as the P*V* is bound if the energy difference between the pair and the isolated defects (i.e. P and *V* being at distances long enough that they do not associate) is negative. The more negative the binding enenrgies the more likely it would be for the P*V* pair to form.

### Modelling *E*-Centres

**Figure 1:**
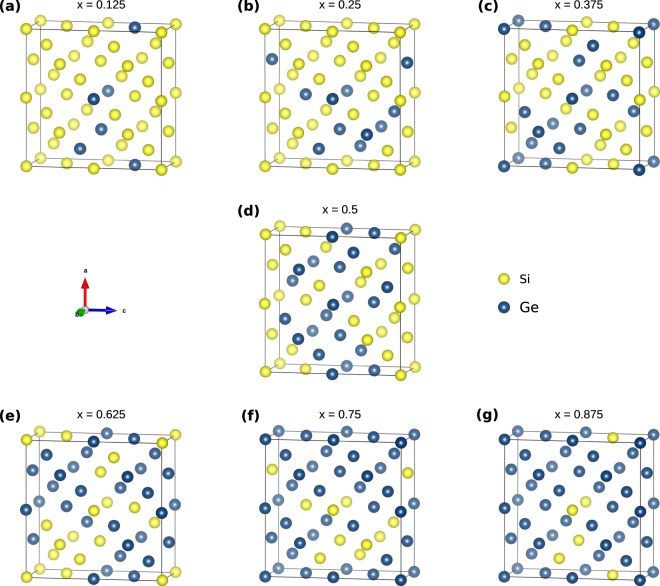
Schematic representation of the 32-atom SQS cells for the Si_1−*x*_Ge_*x*_ (x = 0.125, 0.25, 0.375, 0.5, 0.625, 0.75, 0.875).

Figure 1 is a schematic representation of the SQS cells for the Si_1−*x*_Ge_*x*_ compositions considered. These have been reported in previous work^29^, however, they have not been employed to systematically investigate the defect processes in Si_1−*x*_Ge_*x*_ alloys. Here to study the binding of the phosphorous substitutional-vacancy defect (P*V* pairs ot *E*-centres) we have calculated the energies of all the different nearest neighbour P*V* combinations in the seven Si_1−*x*_Ge_*x*_ alloys considered. To limit defect-image errors typically introduced in computational modelling small supercells we have constructed supercells consisting of two SQS cells each (i.e. 64 atomic sites). Therefore, the total number of P*V* defects considered were 896, whereas more than 1350 DFT calculations were performed to predict the binding energies. The present approach allows the thorough study of defects for a range of compositions and importantly local environment (i.e. Si-rich or Ge-rich). It is an aim of this investigation to derive trends on the impact of composition and environment around the defect on the energetics of *E*-centres.

**Figure 2:**
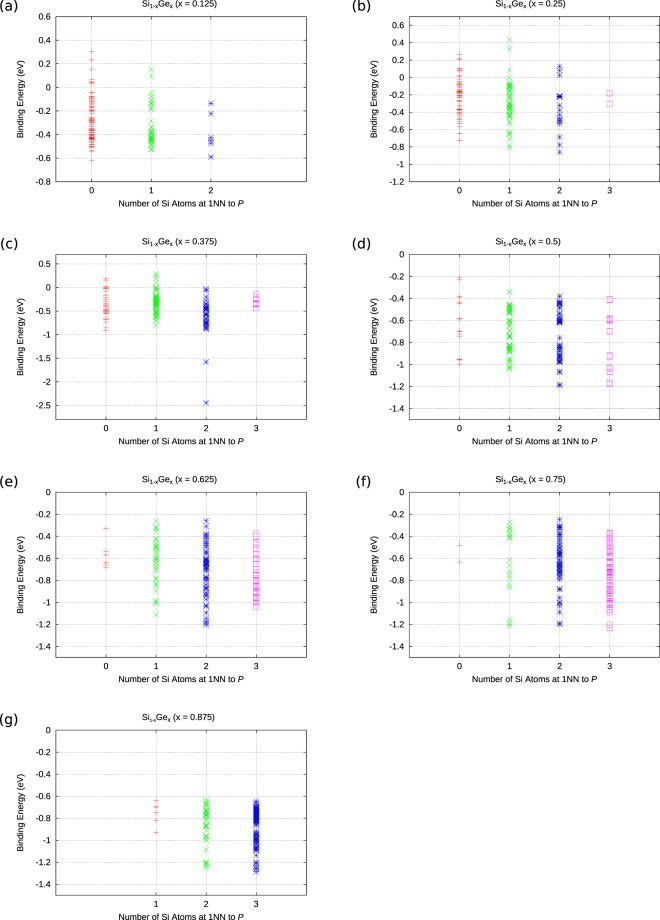
The dependence of the binding energy of P*V* defects in Si_1−*x*_Ge_*x*_ (x = 0.125, 0.25, 0.375, 0.5, 0.625, 0.75, 0.875) with respect to the nearest neighbour atoms to the P atom.

Figure 2 considers the effect of the nearest neighbour host atoms to the P substitutional atom. For Si_1−*x*_Ge_*x*_ cells with up to 62.5% Si content we calculate that there are also positive P*V* binding energies. This implies that P*V* pairs in Si_1−*x*_Ge_*x*_ (x ≤ 0.625) may not form in some areas of the random alloy. This is not the case for Si_1−*x*_Ge_*x*_ (x > 0.625) were the P*V* binding ebergy is always negative (refer to Fig. 2). This is not of course implying that it would be equaly likely for the P*V* to form at any sites in Si_1−*x*_Ge_*x*_ (x > 0.625). The more negative P*V* pairs will be more likely to form and more stable against dissociation.

**Figure 3:**
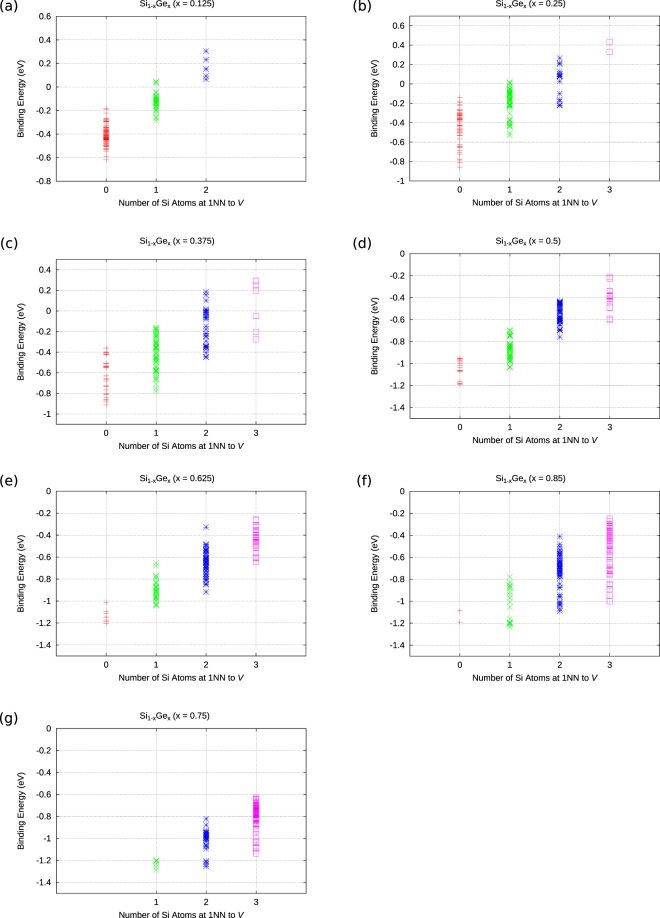
The dependence of the binding energy of P*V* defects in Si_1−*x*_Ge_*x*_ (x = 0.125, 0.25, 0.375, 0.5, 0.625, 0.75, 0.875) with respect to the nearest neighbour atoms to the vacancy.

Figure 3 examines the impact of the first nearest neighbour host atoms to the lattice vacancy. The trend observed is that the *E*-centre will have a more negative binding energy if it forms in the vicinity of Ge atoms, irresppective of the Si_1−*x*_Ge_*x*_ random alloy composition. The physical basis of this can be traced to the lattice relaxation of the Ge host atoms (which are larger than Si atoms) in the vacant space.

**Figure 4:**
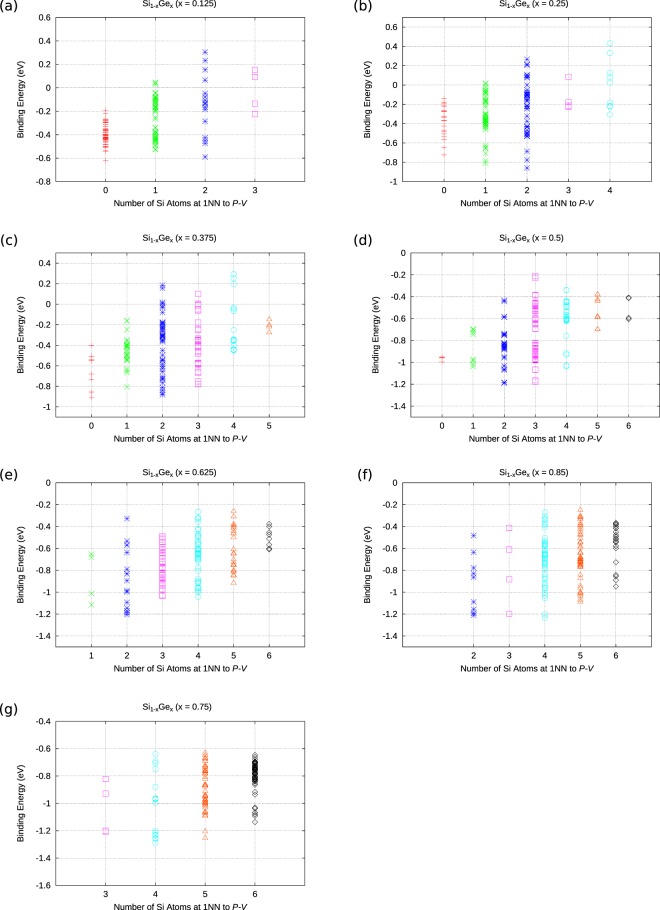
The dependence of the binding energy of P*V* defects in Si_1−*x*_Ge_*x*_ (x = 0.125, 0.25, 0.375, 0.5, 0.625, 0.75, 0.875) with respect to the total nearest neighbour atoms to the P and vacancy.

**Figure 5:**
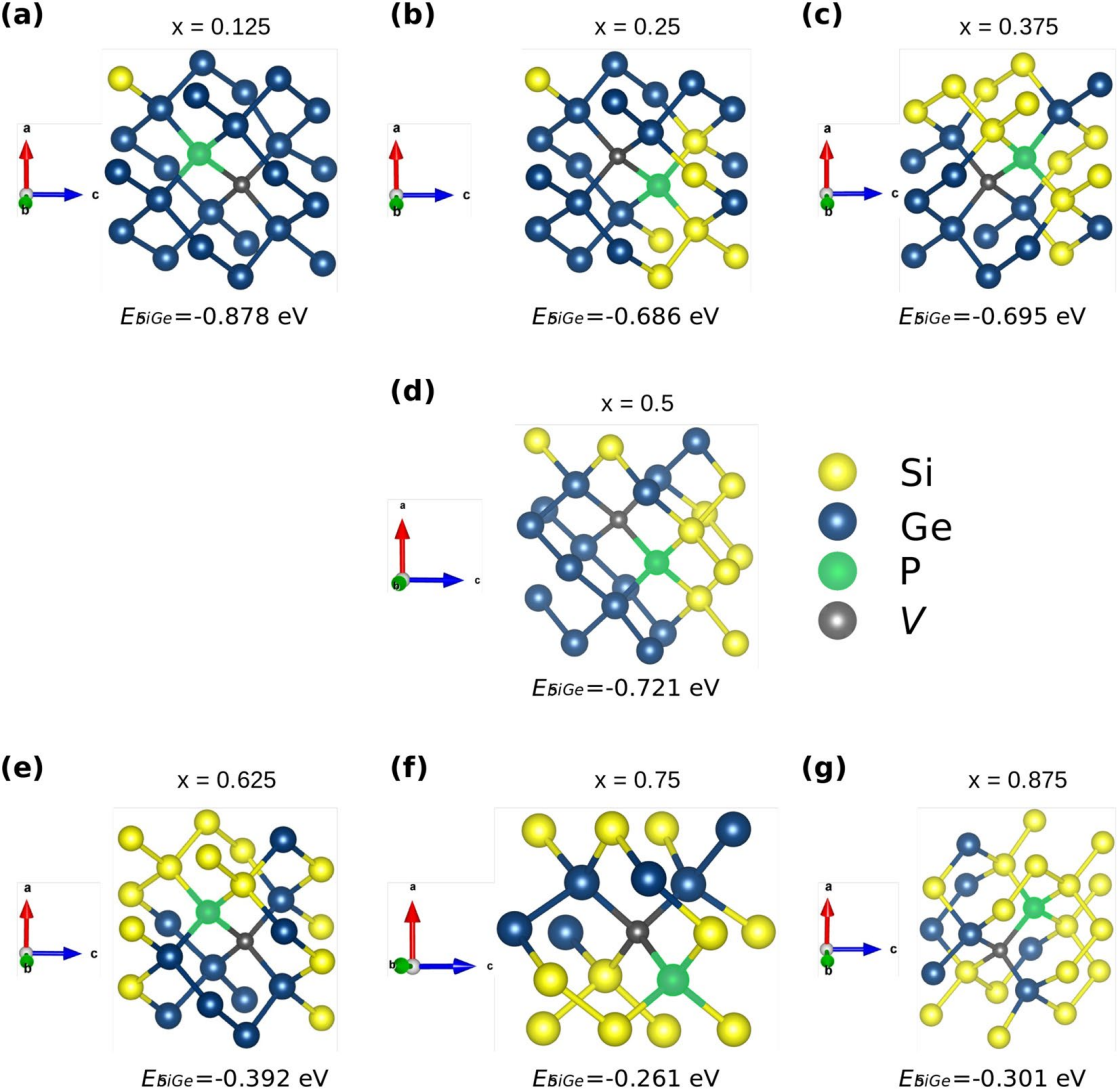
A schematic representation of the lowest binding energy P*V* defects and their nearest neighbour atoms in Si_1−*x*_Ge_*x*_ (x = 0.125, 0.25, 0.375, 0.5, 0.625, 0.75, 0.875).

Figure 4 attempts to sum up the impact of nearest neighbours arounf the *E*-centre. This is to gain an understanding of whether the *E*-centres in Si_1−*x*_Ge_*x*_ prefer to form in Si-rich or Ge-rich regions of the alloy. What can be deduced from Fig. 4 is that *E*-centres form in the vicinity of one or more Ge atoms, irrespective of the alloy composition. For all the alloys considered Ge is strongly represented in the nearest neighbours around the minimum energy P*V*. The most energetically favourable P*V* defects and their nearest neighbour atoms are schematically represented (refer to Fig. 5) for every alloy considered here. The present findings are consistent with previous experimental and theoretical work where it was shown that vacancies preferentially form in the vicinity of Ge atoms^5,25,37^.

**Figure 6:**
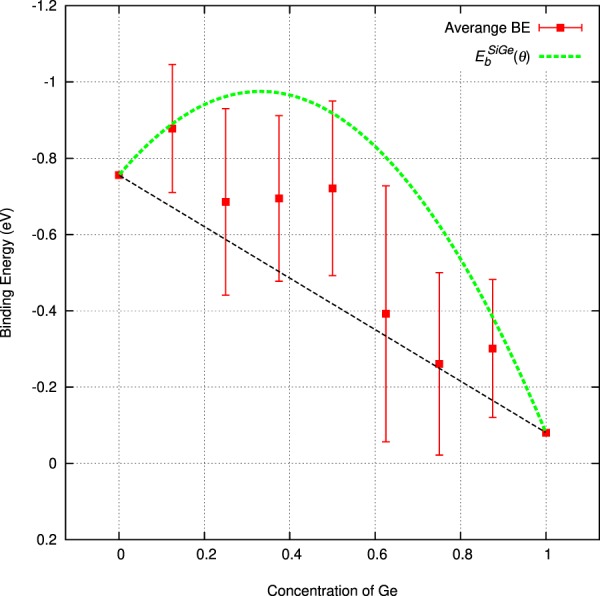
The dependence of the binding energy of P*V* defects with respect to concentration in Si_1−*x*_Ge_*x*_ (x = 0.125, 0.25, 0.375, 0.5, 0.625, 0.75, 0.875).

Figure 6 summarizes the binding energies with respect to alloy composition. The fitted line indicates that binding energies are not linear as a function of alloy composition (i.e. Vegard’s law is invalid in this case). The deviation from linearity of the binding energies of Si_1−*x*_Ge_*x*_ (${E}_{b}^{SiGe}$) as a function of composition can be described via the following relation:

$${E}_{b}^{SiGe}=(1-x){E}_{b}^{Si}+x{E}_{b}^{Ge}+x(1-x)\theta $$

Where θ is called the bowing parameter and is calculated to be −2.0 eV. Although the trend is very similar, this bowing parameter is considerably higher as compared to the one calculated in previous work^5^. This is due to the more substantial SQS cells (32 atom SQS as compared to 16 atom SQS in ref.^5^) and the more Si_1−*x*_Ge_*x*_ compositions used here that allow far more *E*-centre configurations to be considered.

The non-linear dependence of the binding enenrgies with respect to composition is consistent with previous work^5^, however, the present study is far more detailed given more compositions were explored, larger SQS cells and more *E*-centres with richer nearest neighbour environments. What is the nature of this deviation from linearity and is it determined in other defect processes in Si_1−*x*_Ge_*x*_ alloys? The Ge content dependence of the most strongly bound P*V* defects is in agreement with previous experimental studies for donor atom (As and Sb) diffusion in Si_1−*x*_Ge_*x*_ alloys^2,38^. In particular these experimental investigations established that the activation enthalpies of diffusion of As and Sb are not linearly dependent as a function of the Ge content of Si_1−*x*_Ge_*x*_ alloy. More recently, Kube *et al*^3,6^. determined the self-diffusion of Si and Ge in Si_1−*x*_Ge_*x*_ over a wide range of Ge concentration (*x*=0.0, 0.05, 0.25, 0.45 and 0.70) and temperatures (963 K-1543 K). These investigations^3,6^ showed a non-linear dependence of the activation enthalpy of self-diffusion with Ge concentration with a bowing that is consistent with the present study. Thereafter, Saltas *et al*^11^. analysed the experimental results within the c*BΩ* thermodynamic model. In this study, Saltas *et al*^11^. used the thermoelastic properties (bulk modulus, mean atomic volume and thermal expansion coefficient) of Si and Ge to study the composition and temperature dependence of self-diffusion in Si_1−*x*_Ge_*x*_. The resulting deviations from Vegard’s law were attributed to the diversification of the bulk properties of Si and Ge^11^. This is anticipated to be the reason for the bowing in the binding energies of the P*V* defects with respect to composition calculated in the present study, however, further thermodynamic analysis will need to be performed in future work.

**Figure 7:**
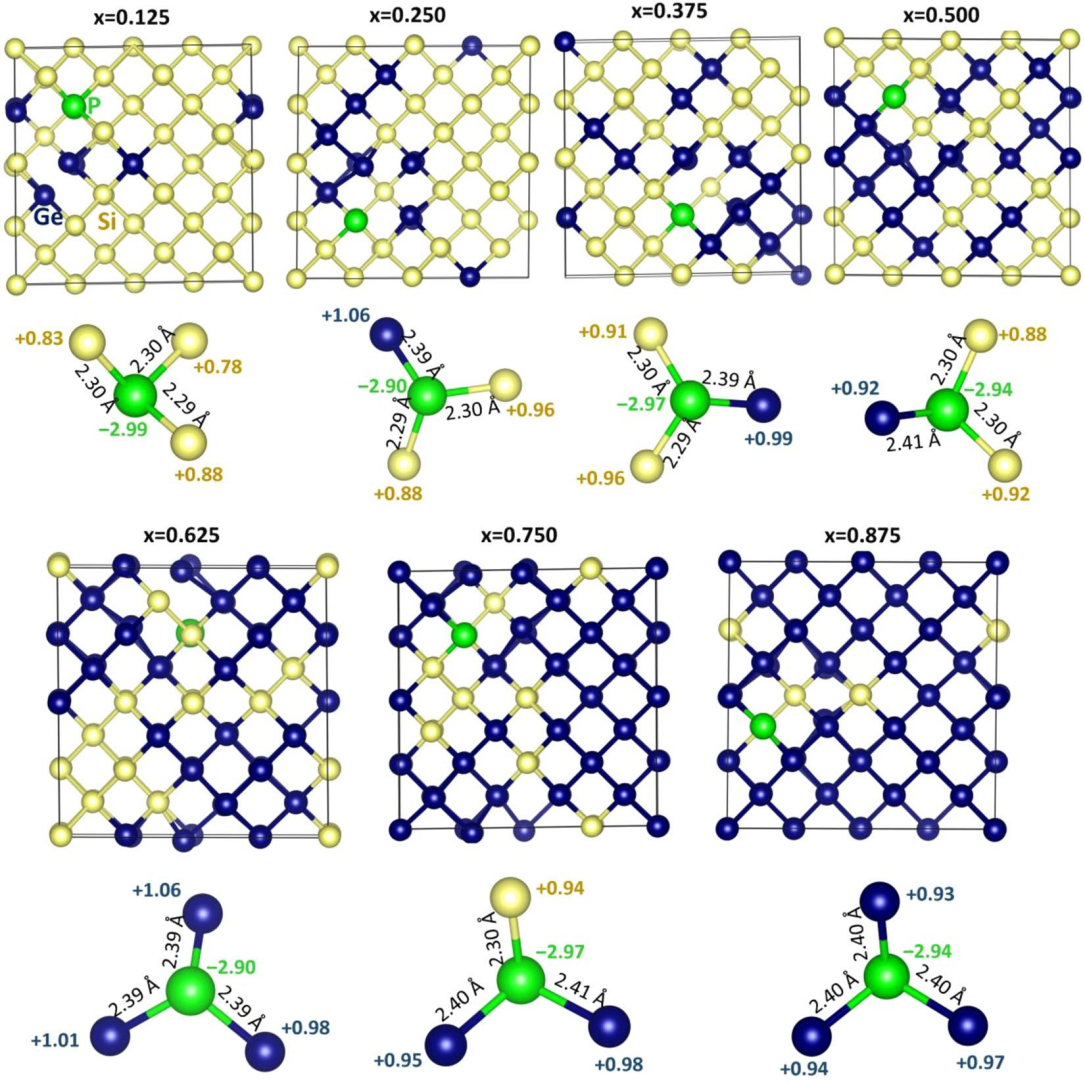
Relaxed structures of seven different phosphorous substitutional-vacancy defect (PV pairs) configurations in Si_1−x_Ge_x_ alloys. A clear view of chemical environment showing bond distances between Si (Ge) and P and Bader charges on P and its nearest neighbour atoms are also reported.

**Figure 8:**
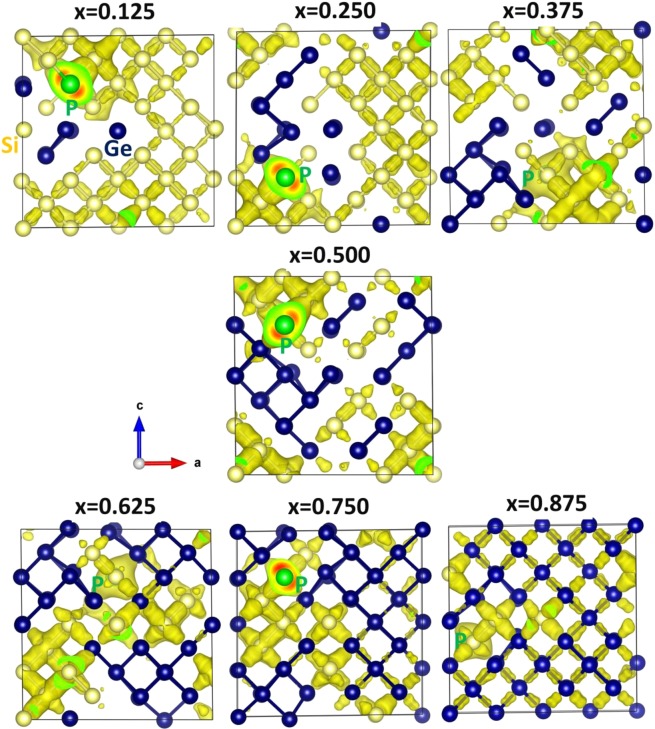
Surface of the constant charge density associated with the interaction of phosphorous with vacancy in each of the seven different configurations in Si_1−x_Ge_x_ alloys.

Apart from relaxation and thermodynamic issues the electronic properties of the defects including charge transfer can impact the properties of Si_1−*x*_Ge_*x*_. To consider this we have performed spin polarized DFT calculations. These calculations enabled us to calculate the amount of charges on the P and its nearest neighbor atoms and plot charge densities localized on the P atoms in each configurations. The relaxed configurations of P interacting vacancies in Si_1−x_Ge_x_ are shown in Fig. 7. In each configurations, there is a substantial interaction between P and Si (or Ge) is observed. This is reflected in the negative Bader charge on P and positive Bader charge on Si (or Ge) (refer to Fig. 8). The amount of charge on P in each configuration is ~−3.00. In each configurations, the P forms three-coordination with adjacent Si (or Ge) atoms and they donate ~1.00 *e* each to P to form stable P^3−^ states. This is further evidenced by the charge density around the P atoms in each configurations (refer to Fig. 8). The Si‒P bond distance is calculated to be ~2.30 Å while the Ge-P bond distance is ~2.40 Å. This is due to the larger atomic radius of Ge than that of Si.

### Summary

In the present study, electronic structure calculations were used to investigate the defect process of the *E*-centre in Si_1−*x*_Ge_*x*_ for a range of compositions. It is shown that the binding energies of the *E*-centres are strongly dependent upon the composition of the alloy, but also on the local environment around the defect. In particular, the more bound *E*-centres have at least one Ge atom as a nearest neighbour, whereas the dependence of the binding energy of the *E*-centres with respect to alloy composition is non-linear. It is shown that the more substantial 32 atom SQS can better describe the defect processes in Si_1−*x*_Ge_*x*_ as compared to 16 atom SQS cells considered previously. The present study is a paradigm of the employment of the SQS method in conjunction with systematic DFT calculations to describe non-linear energetics in random semiconductor alloys. This approach can be extended to other technologically important systems such as random alloys for materials for photovoltaics and solid oxide fuel cells.

### Methods

The binding nature of phosphorous with vacancy defects in Si_1−x_Ge_x_ was examined by using the plane wave DFT code CASTEP^39,40^. The correlation and the exchange interactions are described using the corrected density functional of Perdew, Burke, and Ernzerhof (PBE)^41^, the generalized gradient approximation (GGA), BFGS (Broyden-Fletcher-Goldfarb-Shanno) geometry optimisation algorithm in conjunction with the ultrasoft pseudopotensials^42^. The calculations involved 64-atomic site supercell, the plane wave basis set by choosing the level of convergence of the atomic energies to 0.3 eV/atom, a 2 × 2 × 2 Monkhorst-Pack (MP)^43^ k-point grid. We performed seven sets of calculations for different special quasirandom structures (SQS) configurations for the following concentrations of Si_1−x_Ge_x_ (x = 0.875, 0.750, 0.625, 0.500, 0.375, 0.250, 0.125).

To understand the electronic structures of substitutional phosphorous-vacancy pairs in Si_1−x_Ge_x_ alloys, spin polarized DFT calculations were performed using VASP code^44,45^ which uses plane wave basis sets. The exchange correlation term was modeled using generalized gradient approximation (GGA) parameterized by Perdew, Burke and Ernzerhof^41^. A plane wave basis set with the cut-off energy of 500 eV and a 4 × 4 × 4 Monkhorst-Pack^43^ *k*-point mesh which yields 36 *k* points. Both atom positions and simulation box were relaxed using a conjugate gradient algorithm^46^. Forces on the atoms and stress tensors in all configurations were less than 0.001 eV/Å and 0.002 GPa respectively. Semi-empirical dispersion was included in all calculation as parameterized by Grimme *et al*.^47^ in the VASP code. Bader charge analysis^48^ was used to estimate the charges on the substitutional atom and its nearest neighbours.

## References

[1] Bracht H, Haller EE, Clark-Phelps R (1998). Silicon Self-Diffusion in Isotope Heterostructures. Phys. Rev. Lett..

[2] Laitinen P, Riihimäki I, Räisänen J (2003). & the ISOLDE Collaboration, Arsenic diffusion in relaxed Si_1−*x*_Ge_*x*_. Phys. Rev. B.

[3] Kube R (2008). Simultaneous diffusion of Si and Ge in isotopically controlled Si_1−x_Ge_*x*_ heterostructures. Mater. Sci. Semicond. Process..

[4] Hüger E., Tietze U., Lott D., Bracht H., Bougeard D., Haller E. E., Schmidt H. (2008). Self-diffusion in germanium isotope multilayers at low temperatures. Applied Physics Letters.

[5] Chroneos A, Bracht H, Jiang C, Uberuaga BP, Grimes RW (2008). Nonlinear stability of E centers in Si_1−*x*_Ge_*x*_: Electronic structure calculations. Phys. Rev. B.

[6] Kube R (2010). Composition dependence of Si and Ge diffusion in relaxed Si_1−*x*_Ge_*x*_ alloys. J. Appl. Phys..

[7] Kilpeläinen S (2010). Stabilization of Ge-rich defect complexes originating from *E* centers in Si_1−*x*_Ge_*x*_:P. Phys. Rev. B.

[8] Chroneos A, Bracht H (2014). Diffusion of *n*-type dopants in germanium. Appl. Phys. Rev..

[9] Littlejohns CG (2015). Next generation device grade silicon-germanium on insulator. Sci. Rep..

[10] Prucnal S (2016). Ultra-doped *n*-type germanium thin films for sensing in the mid-infrared. Sci. Rep..

[11] Saltas V, Chroneos A, Vallianatos F (2017). Composition and temperature dependence of self-diffusion in Si_1−x_Ge_x_ alloys. Sci. Rep..

[12] Lee J (2014). Unlocking the potential of cation-disordered oxides for rechargeable lithium batteries. Science.

[13] Jay EE, Rushton MJD, Chroneos A, Grimes RW, Kilner JA (2015). Genetics of superionic conductivity in lithium lanthanum titanates. Phys. Chem. Chem. Phys..

[14] Varotsos P, Alexopoulos K (1980). Calculation of diffusion coefficients at any temperature and pressure from a single measurement. I. Self-diffusion. Phys. Rev. B.

[15] Varotsos P, Alexopoulos K (1981). Calculation of diffusion coefficients at any temperature and pressure from a single measurement. II. Heterodiffusion. Phys. Rev. B.

[16] Varotsos, P., Alexopoulos, K. *Thermodynamics of Point Defects and their Relation with the Bulk Properties*, North-Holland, Amsterdam, (1986).

[17] Chroneos A (2016). Connecting point defect parameters with bulk properties to describe diffusion in solids. Appl. Phys. Rev..

[18] Zunger A, Wei SH, Ferreira LG, Bernard JE (1990). Special quasirandom structures. Phys. Rev. Lett..

[19] Jiang C, Wolverton C, Sofo J, Chen LQ, Liu ZK (2004). First-principles study of binary bcc alloys using special quasirandom structures. Phys. Rev. B.

[20] Chroneos A, Jiang C, Grimes RW, Schwingenschlögl U, Bracht H (2009). E centers in ternary Si_1−*x*−*y*_Ge_*x*_Sn_y_ random alloys. Appl. Phys. Lett..

[21] Jiang C, Stanek CR, Sickafus KE, Uberuaga BP (2009). First-principles prediction of disordering tendencies in pyrochlore oxides. Phys. Rev. B.

[22] Murphy ST, Chroneos A, Grimes RW, Jiang C, Schwingenschlögl U (2011). Phase stability and the arsenic vacancy defect in In_x_Ga_1−x_As. Phys. Rev. B.

[23] Jiang C, Chroneos A (2018). Ab initio modeling of MAX phase solid solutiona using the special quasirandom structure approach. Phys. Chem. Chem. Phys..

[24] Zangenberg NR, Lundsgaard Hansen J, Fage-Pedersen J, Nylandsted Larsen A (2001). Ge Self-Diffusion in Epitaxial Si_1−*x*_Ge_*x*_ Layers. Phys. Rev. Lett..

[25] Venezuela P, Dalpian GM, da Silva AJR, Fazzio A (2002). Vacancy-mediated diffusion in disordered alloys: Ge self-diffusion in Si_1−*x*_Ge_*x*_. Phys. Rev. B.

[26] Dalpian GM, Venezuela P, da Silva AJR, Fazzio A (2002). Ab initio calculations of vacancies in Si_*x*_Ge_1−*x*_. Appl. Phys. Lett..

[27] Laitinen P (2002). Self-Diffusion of ^31^Si and ^71^Ge in Relaxed Si_0.20_Ge_0.80_ Layers. Phys. Rev. Lett..

[28] Chroneos A, Jiang C, Grimes RW, Schwingenschlögl U, Bracht H (2009). Defect interactions in Sn_1−*x*_Ge_*x*_ random alloys. Appl. Phys. Lett..

[29] Chroneos A, Jiang C, Grimes RW, Schwingenschlögl U (2010). Special quasirandom structures for binary/ternary group IV random alloys. Chem. Phys. Lett..

[30] Wang H, Chroneos A, Schwingenschlögl U (2013). Impact of doping on the ionic conductivity of ceria: A comprehensive model. J. Chem. Phys..

[31] Murphy ST, Chroneos A, Jiang C, Schwingenschlögl U, Grimes RW (2010). Deviations from Vegard’s law in ternary III-V alloys. Phys. Rev. B.

[32] Nylandsted Larsen A, Larsen KK, Andersen PE (1993). Heavy doping effects in the diffusion of group IV and V impurities in silicon. J. Appl. Phys..

[33] Ranki V, Nissilä J, Saarinen K (2002). Formation of vacancy-impurity complexes by kinetic processes in highly As-doped Si. Phys. Rev. Lett..

[34] Markevich VP (2004). Vacancy—group V impurity atom pairs in Ge crystals doped with P, As, Sb, and Bi. Phys. Rev. B.

[35] Chroneos A (2006). Implantation and diffusion of phosphorous in germanium. Mater. Sci. Semicond. Process..

[36] Coutinho J (2006). Donor-vacancy complexes in Ge: Cluster and supercell calculations. Phys. Rev. B.

[37] Sihto SL (2003). Vacancy-phosphorus complexes in strained Si_1−*x*_Ge_*x*_: Structure and stability. Phys. Rev. B.

[38] Nylandsted-Larsen A, Kringbøj P (1996). Diffusion of Sb in relaxed Si_1−*x*_Ge_*x*_. Appl. Phys. Lett..

[39] Payne MC, Teter MP, Allan DC, Arias TA, Joannopoulos JD (1992). Iterative minimization techniques for *ab initio* total-energy calculations: molecular dynamics and conjugate gradients. Rev. Mod. Phys..

[40] Segall MD (2002). First-principles simulation: ideas, illustrations and the CASTEP code. J. Phys. Condens. Matter.

[41] Perdew J, Burke K, Ernzerhof M (1996). Generalized gradient approximation made simple. Phys. Rev. Lett..

[42] Vanderbilt D (1990). Soft self-consistent pseudopotentials in a generalized eigenvalue formalism. Phys. Rev. B.

[43] Monkhorst HJ, Pack JD (1976). Special points for Brillouin-zone integrations. Phys. Rev. B.

[44] Kresse G, Furthmüller J (1996). Efficient iterative schemes for ab initio total-energy calculations using a plane-wave basis set. Phys. Rev. B.

[45] Kresse G, Joubert D (1999). From ultrasoft pseudopotentials to the projector augmented-wave method. Phys. Rev. B.

[46] Press, W. H., Flannery, B. P., Teukolsky, S. A. & Vetterling, W. T. Numerical Recipes. The Art of Scientific Computing, Cambridge Univ. Press, Cambridge, 818 pp (1986).

[47] Grimme S, Antony J, Ehrlich S, Krieg H (2010). A consistent and accurate ab initio parametrization of density functional dispersion correction (DFT-D) for the 94 elements H-Pu. J. Chem. Phys.

[48] Henkelman, G., Arnaldsson, A. & Jónsson, H. A fast and robust algorithm for Bader decomposition of charge density. *Comput*. *Mater*. *Sci*. **36**, 354–360, 2005.04.010 (2006).

